# Case of Intra-pelvic Tumor Impeding Delivery

**Published:** 1849-08

**Authors:** Samuel Lewis

**Affiliations:** Philadelphia


### *Case of Intra-pelvic Tumor impeding Delivery.* By Samuel Lewis, M. D., Philadelphia.

I was requested by Mr. T. on the 4th ult., about eleven o'clock, A. M., to see his wife, who was in labor with her second child. On arriving at the house, I learned that labor-pains had commenced early that morning, and had been regular and of increasing strength ever since. After some little time, I proceeded to make the usual examination. I found the os uteri dilated to the size of a quarter of a dollar, with the membranes presenting, and the vagina well lubricated. On passing my finger over the posterior parietes of the vagina, I perceived an unusual fulness, which I took to be the rectum very much loaded. I ordered an enema, and as my presence was not immediately required, I left, promising to call again at one o'clock. At my second visit, I was informed that the injection had been administered, but with very little effect, and that she was unable to pass her urine. I drew off the water, and then made another examination. I found the same state of things, except that the os was further dilated,—there was no diminution, whatever, of the fulness which I had previously observed. Still thinking that it was caused by hardened fæces, I ordered the injection to be repeated ; this was done, but with no better result than the first. I then made an examination per rectum, when I perceived that what I had taken for impacted fæces was a large tumor, occupying the hollow of the sacrum, and situated between the rectum and vagina. It was incompressible, and could not, by any effort that I could make, be displaced. After this examination, I was convinced that delivery could not be accomplished by the natural efforts, until the tumor had been either reduced in its bulk, or pushed up above the brim of the pelvis. As I could not displace it, and as it was impossible for me to decide with any degree of certainty on its nature or contents, I determined, before taking any further step, to try the effect of puncturing it. Accordingly, I obtained my trocars, and after emptying the bladder, I passed one, through the rectum, into the tumor. On withdrawing the stilette I was much gratified to see that fluid escaped. About eight ounces were drawn off, which upon examination proved to be the gruel-like matter of an atheromatous tumor. In about an hour after the operation, my patient was delivered of a male child, rather under the average size. She recovered without a bad symptom. On the fourteenth day after delivery, I made a careful examination both of the vagina and rectum, but could find no trace of the tumor I may state that Mrs. T. assures me, that except a slight difficulty in passing her fæces, she had no symptom whatever of the presence of the tumor. She is a tall, well-made woman, and has always enjoyed good health

There are many cases of pelvic tumors containing liquid or soft contents, scattered through the pages of the medical journals. One of the most interesting that I can call to mind at this time, is that reported in the Med. Rep. for 1826, by Mr. Jackson. The tumor, which was very large, was situated *behind* the rectum, and so completely filled the sacrum, as only to allow of bringing down the child by the feet with great difficulty. An examination of the rectum after delivery, ascertained the existence of fluctuation between the rectum and coccyx; the tumor was punctured, and six pints of straw-colored fluid drawn off. The patient recovered completely, but experienced much suffering from tenderness of the vertebra, pain of the head, and numbness of the lower extremities. It would appear from these symptoms, that the collection, in this case, was connected with the sacral portion of the spinal cord. Several cases of pelvic tumors containing fluid may be found in the second volume of the Med. Chir. Trans., related by Mr. Park. Dr. Merriman likewise published a paper on the subject, either in the eighth or tenth volume (I forget which) of the same work. For a more detailed account of the subject, I must refer to systematic works on midwifery.

